# Resilience dimensions in health system performance assessments, European Union

**DOI:** 10.2471/BLT.23.291102

**Published:** 2024-05-08

**Authors:** Milena Vainieri, Alessia Caputo, Alessandro Vinci

**Affiliations:** a Management and Health Laboratory, Institute of Management, Scuola Superiore Sant’Anna, Via S. Zeno, 2, 56127, Pisa, Italy.

## Abstract

**Objective:**

To explore the definition and operationalization of resilience in health system performance assessments in European Union countries.

**Methods:**

We conducted multiple empirical case study analyses. We identified relevant cases through a literature review from 2014 to 2023 using Google Scholar and through a snowball technique to retrieve additional information. We included only documents that explicitly mentioned resilience in health system performance assessments. We performed a content analysis to identify common patterns in defining resilience.

**Findings:**

The final sample consisted of six countries: Belgium, Croatia, Czechia, Estonia, Ireland and Italy. Each country adopted a distinct approach to conceptualizing resilience, with countries prioritizing specific aspects based on lessons learnt from the coronavirus disease 2019 (COVID-19) pandemic. Some countries focused on maintaining essential health-care services and protecting vulnerable groups. Other countries prioritized management capacity, staff preparedness, digital health utilization and strengthening of primary health care. Content analysis revealed six resilience definitions derived from the key performance indicators: addressing unmet needs and maintaining outcomes; protecting vulnerable groups; acquiring and using resources; having trained and prepared staff in place; using digital health; and strengthening primary health care.

**Conclusion:**

Integration of resilience into the health profiles of European Union countries preceded its inclusion in national health system performance assessments, the latter of which became more prominent after the COVID-19 pandemic. Variations in interpretations within health system performance assessments reflect differences in indicators and policy responses.

## Résumé

**Objectif:**

Explorer la définition et la mise en œuvre de la résilience dans l'évaluation des performances des systèmes de santé au sein des pays de l'Union européenne.

**Méthodes:**

Nous avons effectué plusieurs analyses d'études de cas empiriques. Nous avons identifié les cas pertinents en procédant à une revue de la littérature publiée entre 2014 et 2023 sur Google Scholar et en appliquant la technique de type «boule de neige» pour obtenir des informations supplémentaires. Nous n'avons retenu que les documents qui mentionnaient explicitement la résilience dans les évaluations des performances des systèmes de santé. Enfin, nous avons examiné les contenus afin de repérer les modèles les plus courants dans la définition de la résilience.

**Résultats:**

L'échantillon final était composé de six pays : la Belgique, la Croatie, l'Estonie, l'Irlande, l'Italie et la République tchèque. Chacun de ces pays a adopté une approche différente dans sa conceptualisation de la résilience, certains ayant donné la priorité à des aspects spécifiques en fonction des leçons qu'ils ont tirées de la pandémie de maladie à coronavirus 2019 (COVID-19). Quelques-uns se sont concentrés sur le maintien des services de santé essentiels et la protection des groupes vulnérables. D'autres ont privilégié les capacités de gestion, la préparation du personnel, l'utilisation des technologies numériques et le renforcement des soins de santé primaires. L'analyse des contenus a révélé six définitions de la résilience dérivées des indicateurs clés de performance : répondre aux besoins non satisfaits et assurer le maintien des résultats ; protéger les groupes vulnérables ; acquérir et exploiter les ressources ; disposer de personnel formé et préparé ; déployer des technologies numériques ; et enfin, consolider les soins de santé primaires.

**Conclusion:**

La résilience figurait dans les profils sanitaires de certains pays de l'Union européenne avant d'être intégrée dans les mécanismes d'évaluation des performances des systèmes de santé nationaux, ayant gagné en importance après la pandémie de COVID-19. Les divergences d'interprétation observées dans le cadre de ces évaluations reflètent les variations au niveau des indicateurs et des solutions politiques.

## Resumen

**Objetivo:**

Explorar la definición y la puesta en práctica de la resiliencia en las evaluaciones del rendimiento de los sistemas sanitarios en los países de la Unión Europea.

**Métodos:**

Se realizaron múltiples análisis de estudios de casos empíricos. Se identificaron casos relevantes mediante una revisión bibliográfica de 2014 a 2023 utilizando Google Scholar y mediante una técnica de bola de nieve para recuperar información adicional. Se incluyeron solo los documentos que mencionaban explícitamente la resiliencia en las evaluaciones del rendimiento del sistema sanitario. Se realizó un análisis de contenido para identificar patrones comunes en la definición de resiliencia.

**Resultados:**

La muestra final estaba formada por seis países: Bélgica, Croacia, Chequia, Estonia, Irlanda e Italia. Cada país adoptó un enfoque distinto para conceptualizar la resiliencia, dando prioridad a aspectos específicos basados en las lecciones aprendidas de la pandemia de la enfermedad por coronavirus de 2019 (COVID-19). Algunos países se centraron en mantener los servicios sanitarios esenciales y proteger a los grupos vulnerables. Otros países priorizaron la capacidad de gestión, la preparación del personal, la utilización de la salud digital y el fortalecimiento de la atención primaria. El análisis de contenido reveló seis definiciones de resiliencia derivadas de los indicadores clave de rendimiento: abordar las necesidades insatisfechas y mantener los resultados; proteger a los grupos vulnerables; adquirir y utilizar recursos; contar con personal capacitado y preparado; utilizar la salud digital; y fortalecer la atención primaria de salud.

**Conclusión:**

La integración de la resiliencia en los perfiles sanitarios de los países de la Unión Europea precedió a su inclusión en las evaluaciones del rendimiento de los sistemas sanitarios nacionales, estas últimas más destacadas tras la pandemia de la COVID-19. Las variaciones en las interpretaciones dentro de las evaluaciones del rendimiento de los sistemas sanitarios reflejan diferencias en los indicadores y las respuestas políticas.

## ملخص

**الغرض:**

استكشاف تعريف وتفعيل المرونة في تقييمات أداء النظام الصحي في دول الاتحاد الأوروبي.

**الطريقة:**

قمنا بإجراء العديد من التحليلات التجريبية لدراسة الحالة. وحددنا الحالات ذات الصلة من خلال مراجعة المنشورات من عام 2014 إلى عام 2023 باستخدام Google Scholar، ومن خلال تقنية كرة الثلج لاسترجاع معلومات إضافية. قمنا فقط بتضمين الوثائق التي ذكرت المرونة بشكل صريح في تقييمات أداء النظام الصحي. وأجرينا تحليل للمحتوى لتحديد الأنماط الشائعة في تعريف المرونة.

**النتائج:**

تكونت العينة النهائية من ست دول: بلجيكا، كرواتيا، والتشيك، وإستونيا، وأيرلندا، وإيطاليا. وانتهجت كل دولة أسلوبًا مختلفا لوضع تصور للمرونة، حيث منحت الدول الأولوية لجوانب محددة استنادا إلى الدروس المستفادة من جائحة مرض فيروس كورونا 2019 (كوفيد 19). وركزت بعض الدول على الحفاظ على خدمات الرعاية الصحية الأساسية، وحماية الفئات المهمشة. منحت دول أخرى الأولوية للقدرة الإدارية، ودرجة استعداد فريق العمل، والاعتماد على الصحة الرقمية، وتعزيز الرعاية الصحية الأولية. كشف تحليل المحتوى عن ستة تعريفات للمرونة مشتقة من مؤشرات الأداء الرئيسية: التعامل مع الاحتياجات غير المحققة والحفاظ على النتائج؛ وحماية الفئات المهمشة؛ والحصول على الموارد واستخدامها؛ وتدريب وإعداد فرق العمل في الموقع؛ واستخدام الصحة الرقمية؛ وتعزيز الرعاية الصحية الأولية.

**الاستنتاج:**

إن دمج المرونة في الجوانب الصحية لدول الاتحاد الأوروبي، قد سبق إدراجها في تقييمات أداء النظام الصحي الوطني، وتلك الأخيرة أصبحت أكثر ظهورًا بعد جائحة كوفيد 19. إن الاختلافات في تفسيرات تقييمات أداء النظام الصحي إنما يعكس الاختلافات في المؤشرات واستجابات السياسات.

## 摘要

**目的:**

探讨在欧盟国家卫生系统绩效评估中对复原力的定义和相关操作流程。

**方法:**

我们开展了多次实证案例研究分析。通过使用谷歌学术 (Google Scholar) 检索 2014 年至 2023 年期间的文献综述，以及采用滚雪球方法检索更多信息，我们找到了许多相关案例。我们仅选取了在卫生系统绩效评估中明确提及了复原力的文件。我们采用内容分析方法确定了定义复原力的常规模式。

**结果:**

最终选取了六个国家作为研究样本：爱尔兰、爱沙尼亚、比利时、捷克、克罗地亚和意大利。每个国家采取了不同的方法来定义复原力，且这些国家根据新型冠状病毒肺炎 (COVID-19) 大流行期间总结的经验教训，针对各项具体情况进行了优先排序。有些国家将维持基本的卫生保健服务和保护弱势群体视为工作重点。其他国家则将管理能力、人员配备、数字医疗的利用和加强初级卫生保健列为优先考虑事项。通过采用内容分析方法，我们发现可根据关键绩效指标推导出六种复原力定义：解决未满足需求和维持成果；保护弱势群体；获取和利用资源；安排经培训的储备人员上岗；利用数字医疗；以及加强初级卫生保健。

**结论:**

最初是将复原力纳入了欧盟国家的卫生系统概况，之后又将其纳入了国家卫生系统绩效评估，后者在COVID-19 大流行后变得尤为重要。卫生系统绩效评估中概念解释的差异反映了指标和政策响应情况存在差异。

## Резюме

**Цель:**

Изучить определение и практическую реализацию принципа жизнестойкости при оценке эффективности систем здравоохранения в странах Европейского союза.

**Методы:**

Было проведено несколько эмпирических анализов конкретных случаев. Для поиска релевантных случаев был проведен обзор литературы с 2014 по 2023 год с помощью Google Scholar, а также был использован метод формирования выборки для получения дополнительной информации. При оценке эффективности системы здравоохранения учитывались только те документы, в которых прямо упоминалась жизнестойкость. Для выявления общих закономерностей в определении жизнестойкости был проведен контент-анализ.

**Результаты:**

В окончательную выборку вошли шесть стран: Бельгия, Ирландия, Италия, Хорватия, Чехия и Эстония. В каждой стране был принят свой подход к пониманию жизнестойкости системы, при этом страны определили приоритетность конкретных аспектов на основе уроков, извлеченных из пандемии коронавирусной инфекции 2019 года (COVID-19). Некоторые страны сосредоточились на сохранении основных услуг здравоохранения и защите уязвимых групп населения. Другие страны уделяли первоочередное внимание управленческому потенциалу, готовности персонала, использованию цифровых технологий в здравоохранении и укреплению службы оказания первичной медико-санитарной помощи. По результатам контент-анализа было выявлено шесть определений жизнестойкости, полученных на основе ключевых показателей эффективности: удовлетворение неудовлетворенных потребностей и поддержание результатов, защита уязвимых групп, приобретение и использование ресурсов, наличие обученного и подготовленного персонала, использование цифрового здравоохранения, укрепление службы оказания первичной медико-санитарной помощи.

**Вывод:**

Включение понятия «жизнестойкость» в профили здравоохранения стран Европейского союза предшествовало его включению в оценки эффективности национальных систем здравоохранения, которые стали более заметными после пандемии COVID-19. Различия в интерпретации оценок эффективности системы здравоохранения отражают различия в показателях и политических мерах реагирования.

## Introduction

The coronavirus disease 2019 (COVID-19) pandemic posed a considerable challenge for governments, affecting health, the economy and citizens’ well-being.^1^ The pandemic exposed weaknesses in health systems, such as insufficient workforce capacity^2^ and critical care resources.^3^ This crisis highlighted the necessity for a resilience-centred approach to equip health systems to deal with a wider spectrum of future shocks,^2^ particularly given the varying levels of preparedness among countries.^4^^,^^5^ Failing to prepare for a shock may result in costly interventions with lasting repercussions.^3^ In some cases, these repercussions can permanently alter the status quo of health-care systems, creating a legacy of new challenges. Systems measuring performance, such as health system performance assessments, can support governments in evaluating preparedness, shock management and capacity-building for learning and recovery.^6^ Resilience can be seen as a cross-cutting dimension of the intermediate and ultimate goals of health systems, as well as a factor influencing the performance of the health system.^7^

Although broad consensus exists on the need to bolster health system resilience, questions persist about a shared definition and vision.^8^ One proposal stated that resilience evaluates a system’s ability to maintain performance under major stresses.^9^ The World Bank added other elements by calling for health systems to be alert to threats, responsive to evolving needs, adaptable to minimize disruptions, and capable of post-crisis transformation based on lessons learnt.^10^ The definition used in our study is the one proposed by the European Union (EU) expert panel on effective ways of investing in health.^11^ The panel defined resilience as “the capacity of a health system to (a) proactively foresee, (b) absorb, and (c) adapt to shocks and structural changes in a way that allows it to (i) sustain required operations, (ii) resume optimal performance as quickly as possible, (iii) transform its structure and functions to strengthen the system, and (possibly) (iv) reduce its vulnerability to similar shocks and structural changes in the future.”^11^

Resilience has been measured with different tools such as the United States Agency for International Development (USAID) tool, the World Health Organization (WHO) Joint External Evaluation tool, the Global Health Security Index, or the proposed resilience index,^9^^,^^12^^–^^14^ as well as numerous dashboards set up during the COVID-19 emergency to provide real-time data.^15^ Some of these tools are based on standalone systems using key informant surveys or ad hoc and temporary surveillance data, with limited use of established health information systems. In contrast to this specialized approach, scholars have suggested introducing resilience into a broader framework for health system performance assessment,^7^^,^^9^ especially after the 2013–2016 Ebola virus disease outbreak in West Africa.^8^^,^^16^ Such a framework allows resilience to be monitored within a comprehensive assessment of health system performance. Given that the objectives and functions of health system performance assessment may vary over time, this assessment should be flexible and adaptable.^6^ Moreover, no single universal approach exists that suits every system.^17^ The health system performance assessment can be seen as a country-owned, participatory process that allows the health system to be assessed as a whole and linked to national health strategies whenever possible.^18^^,^^19^ Although the overarching objectives in different countries are the same, such as the improvement of population health, strategic accountability for health system actions needs to be strengthened and policy-makers and other stakeholders should be engaged in articulating health system objectives and priorities. In this way, actions can be harmonized, progress in attainment of goals gauged and informed decision-making stimulated.^20^^,^^21^

Despite numerous attempts to conceptualize resilience, many efforts have remained at the theoretical level. Therefore, we aimed to investigate how countries are putting resilience into practice by measuring key performance indicators, in effect demonstrating which priority areas are considered essential components of each country’s concept of resilience.

## Methods

We performed empirical case study analyses to explore how different countries have integrated the concept of resilience into their health system performance assessment frameworks. At the same time, we investigated whether countries have developed any measurable criteria for assessing resilience.

The case study selection process followed a systematic approach. First, we limited the scope of the analysis to the EU Member States and investigated health system performance assessments to countries with available data. We chose to limit our analysis to the EU for several reasons. First, the EU has played a central role in endorsing initiatives on health system performance assessment since 2014, when it established an expert group on health system performance assessment^21^ to facilitate knowledge exchange among member states.^22^ Additionally, the European Commission, through the technical support instrument,^23^ has assisted health authorities in different countries in implementing health system performance assessment frameworks tailored to the country. Second, publicly disclosing health system information is standard practice in the EU through initiatives such as the European Commission’s biennial country health profiles, which also include resilience measures.^24^ Thus, a supranational organization already exists that guides the incorporation of resilience into health system performance assessment. Finally, with the EU moving towards a European Health Union,^25^ which aims to facilitate health-care delivery across internal EU borders,^26^ a culture of coordination is growing within the union, extending partially to the health-care sector, despite member states retaining sovereignty over health care.^26^

We conducted a literature review using Google Scholar to identify relevant documents drawn from scientific and grey literature sources published from January 2014 to February 2024, with no language restrictions applied. We chose Google Scholar because of its extensive coverage of peer-reviewed articles, books, conference papers and other reports, including official and institutional documents. We selected 2014 as a reference year because it marked the global recognition of resilience in health-care systems after the Ebola virus disease outbreak. In addition, the European Commission emphasized the importance of resilience in its 2014 publication *Communication from the Commission on effective, accessible and resilient health systems*.^27^

After screening the titles and abstracts of the documents, we integrated the initial sample with information drawn from the websites of health ministries, health boards and health agencies. We examined websites and reports of international organizations, with attention to the countries that received support from the European Commission in developing their health system performance assessment.^23^ We took this precautionary step to prevent the inadvertent omission of these countries as their national authorities may have not yet published any information, possibly due to ongoing programme activities. We identified additional articles and reports using a snowball technique,^27^ starting with the references of seminal studies.

We excluded documents that did not focus on assessment of national health system performance; additionally, we excluded publications that examined specific health-care topics without adopting a comprehensive system-wide perspective. We selected only those health system performance assessments where the resilience dimension was reported (14 documents). In the chosen case studies, we performed a content analysis,^28^^,^^29^ examining all pertinent documents and websites for the countries starting from the first year resilience was explicitly introduced. In our content analysis, we used a deductive approach^30^ to measure resilience, based on the definition provided by the EU expert panel on effective ways of investing in health.^11^ We initially categorized the key performance indicators documented within the health system performance assessments of the countries in line with the four points outlined in the EU expert panel’s definition of resilience, namely: (i) sustaining operations; (ii) resuming optimal performance swiftly; (iii) transforming structure and functions to strengthen the system; and (iv) reducing vulnerability to future shocks.^11^ After expanding on these four points, we were able to better classify the concept of resilience through the way the key performance indicators operationalized resilience.

## Results

**Fig. 1:**
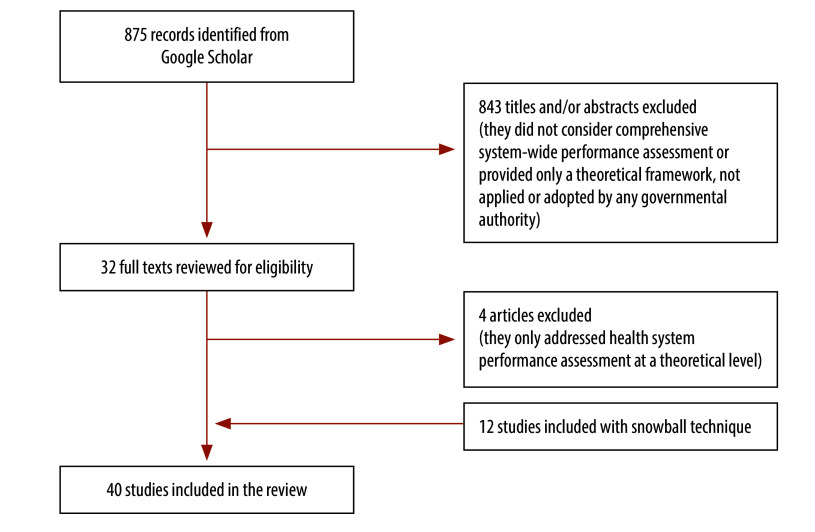
Flowchart of selection of documents on national health system performance assessments, European Union

We retrieved 886 documents with the review of the literature and the snowball procedure. We excluded most of these documents as they considered health system performance assessment only at a theoretical level without analysing country-owned health system performance assessments. The final sample comprised 40 documents, excluding repeated references, websites and platforms (Fig. 1 and Table 1; Albreht et al., unpublished report, 2023).^17^^,^^31^^–^^68^

**Table 1:** References for each country for health system performance assessment, European Union

Country	First year of assessment	Sources
Austria	2013	Bachner et al., 2018^31^ Bachner et al., 2018^32^
Belgium	2012	European Commission, 2016^17^ Devos et al., 2019^33^ Gerkens et al., 2020^34^ Gerkens et al., 2023^35^
Bulgaria	Not a comprehensive health system performance assessment	Rohova et al., 2017^36^ Dimova et al., 2018^37^
Croatia	2023	Sagan et al., 2021^38^ Croatian Ministry of Health, 2023^39^ Albreht et al., unpublished report, 2023 (available on request from the corresponding author)
Cyprus	NA	NA
Czechia	Ongoing process (expected in 2025)	Bryndová et al., 2023^40^ OECD, 2023^41^
Denmark	NA	NA
Estonia	2023	OECD, 2023^42^
Finland	2004	European Commission, 2016^17^ Kilpeläinen et al., 2016^43^ Keskimäki et al., 2019^44^ National websites and platforms
France	2004	European Commission, 2016^17^ Or et al., 2023^45^ Autorité de Santé, 2024^46^ National websites and platforms
Germany	Not a comprehensive health system performance assessment	European Commission, 2016^17^ Blümel et al., 2020^47^ Röttger et al., 2018^48^ National websites and platforms
Greece	NA	NA
Hungary	2016	Szigeti, et al., 2017^49^ Brito Fernandes et al., 2022^50^ National websites and platforms
Ireland	2023	Brito Fernandes et al., 2021^51^ Kringos et al., 2021^52^ Kringos et al., 2021^53^ Government of Ireland Department of Health, 2023^54^ National websites and platforms
Italy	2008	European Commission; 2016^17^ Vola et al., 2022^55^ Vainieri & Vola, 2023^56^ Regional websites and platforms
Latvia	2019	Noto et al., 2019^57^ Brigis et al., 2020^58^ Albreht et al., unpublished report, 2023 (available on request from the corresponding author)
Lithuania	2019^a^	NA
Luxembourg	NA	NA
Malta	2014	Azzopardi Muscat et al., 2014^59^ Grech et al., 2015^60^ European Commission, 2016^17^ Grech, 2018^61^
Netherlands, Kingdom of the	2006	Van den Berg et al., 2014^62^ Van den Berg et al., 2014^63^
Poland	NA	NA
Portugal	2009	European Commission, 2016^17^ de Almeida Simões et al., 2017^64^
Romania	NA	Cojoaca et al., 2022^65^ Vladescu et al., 2010^66^
Slovakia	NA	NA
Slovenia	2019	Perko et al., 2019^67^
Spain	2003	Spain Ministry of Health, 2022^68^
Sweden	2003	European Commission, 2016^17^

EU: European Union; NA: not available; OECD: Organisation for Economic Cooperation and Development.

^a^ Estimation based on the EU support programme.

Table 2 summarizes EU countries’ population, economic and health system profiles. We identified 18 countries that had adopted comprehensive system-wide health system performance assessments. Seven countries explicitly included resilience: Belgium, Croatia, Czechia, Estonia, Ireland, Italy and Lithuania. In Italy, as a decentralized system, we identified and considered the health system performance evaluations conducted at the regional level of government. Four countries had EU support in their health system performance assessments. This limited number of countries may be due to the slow reaction and inertia of complex organizations to change.^56^

**Table 2:** Country profiles and national health system performance assessments, European Union

Country	Country profile						Health system performance assessment		
	Population size^a^	% of population aged > 65 years^a^	GDP per capita, PPP^a,b^	Health expenditure, US$ per capita^c^	Health-care system		Assessment retrieved	EU support	Included section on resilience
Austria	8 978 929	19.4	44 065	5585	Mixed model		Yes	No	No
Belgium	11 617 623	19.5	42 213	5009	Social health insurance		Yes	No	Yes
Bulgaria	6 838 937	21.7	20 709	857	Social health insurance		No	No	No
Croatia	3 862 305	22.5	25 732	1095	Social health insurance		Yes	Yes	Yes
Cyprus	904 705	16.5	32 349	2245	National health system		No	No	No
Czechia	10 516 707	20.6	31 953	2120	Social health insurance		Yes	Yes	Yes
Denmark	5 873 420	20.3	48 114	6438	National health system		No	No	No
Estonia	1 331 796	20.4	30 671	1788	Social health insurance		Yes	Yes	Yes
Finland	5 548 241	23.1	38 679	4726	National health system		Yes	No	No
France	67 871 925	21.0	35 769	4769	Social health insurance		Yes	No	No
Germany	88 237 124	22.1	41 246	5930	Social health insurance		No	No	No
Greece	10 459 782	22.7	23 934	1675	Social health insurance		No	No	No
Hungary	9 689 010	20.5	27 259	1163	Social health insurance		Yes	No	No
Ireland	5 060 004	15.0	82 100	6092	National health system		Yes	Yes	Yes
Italy	59 030 133	23.8	33 688	3057	National health system		Yes	No	Yes
Latvia	1 875 757	20.9	25 939	1313	Social health insurance		Yes	Yes	No
Lithuania	2 805 998	20.0	31 481	1522	Social health insurance		Yes	Yes	Yes
Luxembourg	645 397	14.8	91 870	6757	Social health insurance		No	No	No
Malta	520 971	19.2	35 992	3135	National health system		Yes	No	No
Netherlands, Kingdom of the	17 590 673	20.0	46 093	5846	Social health insurance		Yes	No	No
Poland	37 654 247	19.1	28 044	1026	Social health insurance		No	No	No
Portugal	10 352 042	23.7	27 237	2342	National health system		Yes	No	No
Romania	19 042 455	19.5	27 073	810	National health system		No	No	No
Slovakia	5 234 712	17.4	24 061	1394	Social health insurance		No	No	No
Slovenia	2 107 180	21.1	32 546	2417	Social health insurance		Yes	Yes	No
Spain	47 432 893	20.1	29 808	2901	National health system		Yes	No	No
Sweden	10 452 326	20.3	42 264	6028	National health system		Yes	No	No

EU: European Union; GDP: gross domestic product; PPP: purchasing power parity; US$: United States dollars; WHO: World Health Organization.

^a^ Sources: (i) WHO, 2023^69^ (ii) European Commission, 2021.^70^

^b^ GDP per capita is expressed in euros, adjusted by PPP.

^c^ Source: WHO, 2023.^71^

Table 3 outlines how health system resilience is conceptualized and operationalized into key performance indicators across six of the seven countries. We omitted Lithuania due to the unavailability of documents that could elucidate how resilience had been interpreted. Italy is repeated in the table due to the periodic release of its health system performance assessments. As highlighted in the year column of Table 3, health system performance assessment frameworks explicitly incorporating the resilience dimension predominantly emerge in updates after 2019, reflecting a trend after the COVID-19 pandemic.

**Table 3:** Domain of resilience in national health system performance assessments, Belgium, Croatia, Czechia, Estonia, Ireland, Italy and Lithuania

Country	Year	Resilience		
		Short definition	Dimension in the health system performance assessment framework	Type of key performance indicators considered
Belgium	2023	Health system capacity to proactively foresee, absorb and adapt to shocks, only in relation to the COVID-19 pandemic	Yes	Human resource indicators (e.g. intention to leave by professionals, shortages); capacity to provide services; efficiency in acute care beds management; use of digital health; prompt response to COVID-19 testing; and % COVID-19 vaccination coverage
Croatia	2023	Capacity to respond to shocks and enhance efficiency amid growing health-care demands with constrained resources. This definition emphasizes structure-related investments	Yes	Capital expenditure; generic pharmaceutical usage; ambulatory surgery; medical and nursing workforce; public health and long-term care expenses; and diagnostic imaging procedures
Czechia	Forthcoming	Ability of the health system to absorb, respond to and adapt to unexpected events	Yes	Mental health care; early detection of drug shortages; primary care capacity; efficiency in acute care beds management; and number of beds for acute care and long-term care per inhabitant
Estonia	2023	Capacity to proactively adapt and quickly respond to challenges ensuring resilience, continuity and quality of service delivery	Yes	Preparedness; and vaccination
Ireland	2023	Adaptability of the national health system in response to diverse situations and needs, primarily focusing on its capacity and workforce motivation and support	Yes	Health worker job satisfaction; health worker absenteeism; use of staff support mechanisms (e.g. helplines); and surge capacity; capacity to scale up and down resources and volumes of services
Italy	2020	Capacity to ensure the service and resume optimal performance as quickly as possible	Yes	Compared with the last pre-pandemic year, differences in volumes for a selected list of: oncological treatments; outpatients visits; and drugs and primary health-care services
Italy	2021	Capacity to ensure the service, resume optimal performance as quickly as possible and reduce health system vulnerability	Yes	Same indicators as 2020 above, plus vaccination against COVID-19 and use of digital health
Italy	2022	A more systematic approach was adopted based on 2020 and 2021 definitions to ensure the system is ready to face future crises	No specific domain but reported as indicators to measure preparedness	Vaccination coverage of fragile groups; residential and long-term care; health worker absenteeism; mental health; and digital health

COVID-19: coronavirus disease 2019.

Table 4 summarizes our content analysis, which revealed six resilience definitions derived from the key performance indicators in selected health system performance assessments. These definitions are compared with the EU expert panel’s theoretical four-category definition.^11^

**Table 4:** Overlap of theoretical and operational definitions of resilience in national health system performance assessments, Belgium, Croatia, Czechia, Estonia, Ireland, Italy and Lithuania

Operational definitions of resilience	Theoretical definition of resilience^a^			
	Sustain required operations	Resume optimal performance	Transform health-care structure and functions to enhance system strength	Reduce vulnerability to future shocks
Capacity to address unmet needs and maintain outcomes	Yes	Yes	No	No
Capacity to protect vulnerable groups	No	No	No	Yes
Capacity of management to acquire and use resources	No	No	Yes	No
Capacity to have trained and prepared staff in place	Yes	No	Yes	No
Capacity to utilize digital health	Yes	No	Yes	No
Capacity to strengthen primary health-care services	Yes	Yes	Yes	Yes

^a^ Source: European Commission; 2022.^11^

Note: Yes indicates that the operational and theoretical definitions of resilience overlap; no indicates that the operational and theoretical definitions of resilience do not overlap.

### Address unmet needs and maintain outcomes

Three health system performance assessments incorporated key performance indicators related to the ability to maintain essential health services and quickly resume optimal performance, particularly in scenarios involving treatment restrictions or limitations (Italy in 2020 and 2022^55^ and Belgium in 2023;^35^ Table 3). Resilience was defined in relation to the COVID-19 pandemic. Italian regions assessed their capacity to sustain the same level of urgent treatments and follow-up visits to avert potential unmet needs,^55^ while Belgium examined the number of new invasive cancer cases as a direct adverse impact on the health of the population.^35^ This operationalization covers the first two aspects of the definition of resilience proposed by the EU’s expert panel on effective ways of investing in health, namely sustaining required operations and resuming optimal performance.^11^

### Protect vulnerable groups

Belgium (2023),^35^ Estonia (2023)^42^ and Italy (2021)^55^ introduced resilience measures aimed at reducing vulnerabilities to better withstand current and future shocks (Table 3). These countries considered key performance indicators that evaluated reducing health system vulnerability through COVID-19 vaccination coverage. Some national health system performance assessments broadened this concept by including indicators to assess the health system’s ability to protect vulnerable groups through preventive efforts. Estonia (2023)^42^ incorporated indicators measuring seasonal vaccination coverage rates and the incidence of vaccine-preventable diseases per 100 000 population. Italy (2022)^55^ integrated metrics of influenza vaccination coverage for older people and health workers. Czechia (2023)^41^ and Italy (2022)^55^ developed measures that gauge the health system’s capacity to provide mental health services. This operationalization overlaps with the last category of the EU’s expert panel, that is, reducing vulnerability to future shocks.^11^

### Acquire and use resources

A more nuanced conceptualization of resilience emerged in some countries which placed greater emphasis on structure-related elements, which is consistent with the EU’s 2016 and 2018 interpretation of resilience.^72^^,^^73^ Key performance indicators included generic pharmaceutical usage, public health, long-term care expenses and bed occupancy rates (Belgium, 2023);^35^ and average waiting time between tests and results and surge capacity (Ireland, 2023).^54^ Croatia (2023; Albreht et al., unpublished report, available on request from the corresponding author) and Czechia (2023)^41^ focused on the enhancement of investment and the promotion of policy reform, with the aim of strengthening technological and infrastructure capabilities (Table 3). This operationalization overlaps with the third aspect of the definition of resilience of the EU’s expert panel, namely transforming health system structure and functions to enhance the system’s strength.^11^

### Trained and prepared staff

Resilience can also be assessed through measures related to the health-care workforce, as their dedication and well-being are integral to maintaining resilient health-care systems. Indicators can be either quantitative, such as the number of medical doctors and nurses or beds (Belgium 2023,^35^ Czechia 2023^41^ and Ireland 2023);^54^ or qualitative, which focuses on well-being of health professionals. This latter aspect involves evaluating job satisfaction through metrics such as intention to leave and absenteeism, as adopted by Belgium (2023),^35^ Croatia (2023)^39^ and Ireland (2023).^54^ Ireland (2023)^54^ incorporated key performance indicators to monitor specific policies on human resources such as establishing helplines for professionals (Table 3). This operationalization crosses two dimensions of the definition of resilience of the EU’s expert panel, that is, sustaining required operations and transforming health-care structure and functions to enhance the system’s strength.^11^

### Utilize digital health

Some countries have incorporated digital channels into their resilience dimension, driven by the accelerated digitalization of health care in response to the COVID-19 pandemic. Belgium in 2023^35^ and Italy in 2021 and 2022^55^ integrated these supply-side indicators into the health system performance assessments to monitor whether health-care systems succeeded in reducing backlogs and ensuring continuity of care by delivering services through digital channels (Table 3). This operationalization crosses two dimensions of the definition of resilience of the EU’s expert panel, namely, sustaining required operations and transforming its structure and functions to enhance the system’s strength.^11^

### Strengthen primary health care

Resilience is sometimes construed as the capacity to strengthen primary care as a form of preparedness. Significant links existed between the core functions of primary care in non-health emergencies and a country’s ability to effectively respond to and recover from the COVID-19 pandemic.^3^ Some countries incorporated indicators of primary health care into the resilience dimension to highlight the correlation between the capacity to provide care during outbreaks. Indicators related to primary health care often relate to investments in structural aspects, such as long-term care (e.g. Croatia in 2023,^39^ Czechia in 2023^41^ and Italy in 2022).^55^ Some indicators emphasize the reinforcement of access, such as the number of contacts with general practitioners and mental health services provided (e.g. Czechia in 2023^41^ and Italy in 2022);^55^ and vaccination coverage for vulnerable populations (Italy, 2021 and 2022),^55^ which in some instances are included in other dimensions (Belgium, 2023).^35^ In a broader context, all the health system performance assessments examined incorporated primary health-care indicators either within specific domains or as cross-cutting factors. These indicators assess the effectiveness of primary health care through measures such as ambulatory-related conditions, access, coordination and service continuity. Some countries included primary health-care indicators within other dimensions directly tied to resilience, such as expanding the primary health-care workforce and allocating health expenditure to primary care (Ireland^54^ and Croatia^39^ in 2023). Additionally, the catchment index (i.e. the number of visits or diagnostic examinations in relation to those prescribed) contributes to resilience by revealing potential unmet needs (Table 3). This holistic vision of primary health care aligns with the aim of resilience to ensure systems can bounce back, adapt, learn and improve in crises, potentially spanning all dimensions of resilience as defined by the EU’s expert panel on effective ways of investing in health.^11^

## Discussion

We investigated how resilience has been defined and integrated into various European health system performance assessment frameworks. We sought to clarify the conceptual framing of resilience by examining the key performance indicators in these health system performance assessments. Although the EU recommended including resilience in health system performance assessments as early as 2014,^27^ all the countries analysed in our study only incorporated this dimension after the COVID-19 pandemic.

Resilience refers to a health system’s capacity to adapt and maintain control over its structure and functions, even when confronted with significant stresses.^74^ Traditionally, the focus has been on risk-management strategies to prevent and mitigate threats, but the complexities of contemporary systems make this approach insufficient.^75^ The COVID-19 pandemic led to a paradigm shift that acknowledged the unpredictability of systemic threats and emphasized the need to enhance health system resilience.^76^ This perspective highlights the importance of a health system’s ability to anticipate, absorb, recover from and adapt to a wide range of disruptions.^76^^,^^77^ Incorporating resilience as an element within national and regional health system performance assessments^78^ is a practical strategy to enhance the ability of health systems to withstand and recover from disruptions efficiently.

The findings of a recent analysis of health system performance assessments^7^ have been partially integrated by most national health system performance assessments, demonstrating the ability of measurement systems to adapt to contemporary environments. This adaptation is particularly evident in the case of Italy, where resilience indicators dominated during the pandemic, both in system delivery (e.g. testing for COVID-19 and COVID-19 vaccination) and final goals of the health system (e.g. pandemic mortality rate). In contrast, in non-pandemic periods, key performance indicators of resilience mainly related to primary health care, particularly in the domains of system delivery and intermediary outcomes. This finding aligns with the broader understanding that resilient health-care systems require robust primary health-care foundations^7^ to ensure accessibility, equity and continuity of care, even in the face of unprecedented challenges.^79^^,^^80^ The concept of resilience seems to have evolved, as shown by the changes in the key performance indicators within the Belgian and Italian health system performance assessments. This shift underscores the importance of adaptability in implementing resilience strategies in response to changing environments.^56^

Overall, most countries have adopted a definition of health system resilience that emphasizes the ability to anticipate, absorb and adapt to shocks through the following dimensions: (i) capacity to address unmet needs and maintain outcomes; (ii) capacity to protect vulnerable groups; (iii) capacity of management to acquire and use resources; (iv) capacity to have trained and prepared staff in place; (v) capacity to utilize digital health; and (vi) capacity to strengthen primary health-care services. However, each country has tailored this definition to suit its unique health-care landscape and lessons learnt from the pandemic. Some countries have prioritized maintaining essential health services and quickly resuming optimal performance during a pandemic, while others have focused on reducing vulnerabilities within their health-care system. Our analysis indicates that health system performance assessment frameworks incorporating resilience emerged in updated post-pandemic assessments. Initially, countries with pre-existing health system performance assessments introduced resilience metrics in response to the COVID-19 pandemic with a focus on maintaining essential services during critical phases. Later, these countries assessed the resilience of their health-care systems to future shocks by introducing key performance indicators related to the health workforce, digital health and strengthening of primary health care. In particular, investments in digital technologies, such as digital consultations and telehealth services, can streamline patient pathways, minimizing the need for in-person doctor visits.^3^ While vital for resilience of health-care systems, these investments necessitate integrated information systems and care models to enhance patient-care coordination and decision-making.

The concept of resilience has been addressed to a lesser extent in wider health system performance assessments. This concept has evolved from addressing immediate outbreak responses during the initial phases of a pandemic, to a stronger focus on proactive preparedness measures aimed at mitigating future epidemic impacts through strengthening health-care systems. The implementation and scaling up of these measures depends on the availability of data. Some data, such as using digital health and strengthening primary health care, could be easily collected and included in health system performance assessments. However, other data, such as preparedness measures, are not systematically monitored. Finally, while some key performance indicators may have a broader scope, such as job satisfaction of health workers, they can be classified under resilience. The inclusion of such key indicators as a measure of resilience was identified in the case studies analysed as a predictor of health-care systems’ readiness for future shocks. 

Our study has some limitations. First, our inclusion criteria were restricted to health system performance assessments explicitly mentioning the term resilience. Countries using different terminologies, such as preparedness and responsiveness, were not included. However, the objective of our analysis was to examine resilience and how it was defined and operationalized into key performance indicators. Future studies could investigate how countries adopted different terms to refer to the concept of resilience. Additionally, we limited the geographical scope to the EU, for reasons outlined in the methods section. Nevertheless, it is important to acknowledge global heterogeneity in health system performance assessment frameworks and to provide perspectives beyond Europe. Thus, the study may only partially capture the diversity in health system performance assessments globally.
